# Transcriptome Analysis and Identification of the Cholesterol Side Chain Cleavage Enzyme BbgCYP11A1 From *Bufo bufo gargarizans*

**DOI:** 10.3389/fgene.2022.828877

**Published:** 2022-04-05

**Authors:** Guangli Li, Tianyue An, Yu Li, Jinyang Yue, Ruoshi Huang, Jia Huang, Jincai Liang, Wei Yao, Liufang Huang, Yidu Chen, Rongrong Zhang, Aijia Ji, Lixin Duan

**Affiliations:** ^1^ Joint Laboratory for Translational Cancer Research of Chinese Medicine of the Ministry of Education of the People’s Republic of China, International Institute for Translational Chinese Medicine, School of Pharmaceutical Sciences, Guangzhou University of Chinese Medicine, Guangzhou, China; ^2^ School of Integrated Traditional Chinese and Western Medicine, Binzhou Medical University, Yanta, China

**Keywords:** *Bufo bufo gargarizans*, transcriptome, cholesterol side chain cleavage enzyme, bufadienolides biosynthesis, *Saccharomyces cerevisiae*

## Abstract

*Bufo bufo gargarizans* Cantor are precious medicinal animals in traditional Chinese medicine (TCM). Bufadienolides as the major pharmacological components are generated from the venomous glands of *B. bufo gargarizans*. Bufadienolides are one type of cardiac aglycone with a six-member lactone ring and have properties of antitumor, cardiotonic, tonsillitis, and anti-inflammatory. The biosynthesis of bufadienolides is complex and unclear. This study explored the transcriptome of three different tissues (skin glands, venom glands, and muscles) of *B. bufo gargarizans* by high-throughput sequencing. According to the gene tissue–specific expression profile, 389 candidate genes were predicted possibly participating in the bufadienolides biosynthesis pathway. Then, BbgCYP11A1 was identified as a cholesterol side chain cleaving the enzyme in engineering yeast producing cholesterol. Furthermore, the catalytic activity of BbgCYP11A1 was studied with various redox partners. Interestingly, a plant NADPH-cytochrome P450 reductase (CPR) from *Anemarrhena asphodeloides* showed notably higher production than BbgAdx-2A-BbgAdR from *B. bufo gargarizans*. These results will provide certainly molecular research to reveal the bufadienolides biosynthesis pathway in *B. bufo gargarizans*.

## Introduction

*Bufo bufo gargarizans* Cantor (Bufonidae) are Chinese national protected wild animals with important economic and scientific research value. Chan’Su is a class of dried secretion in the venom gland or skin gland of *Bufo bufo gargarizans* Cantor and is used as an animal medicinal material with a long history. Bufadienolides, one kind of cardiac aglycone with a six-membered lactone ring, are the important bioactive ingredients of Chan’Su. Recent biological studies confirm the significant anticancer activities of bufadienolides *in vitro* and *in vivo* (Nogawa et al., 2001; Watanabe et al., 2003; Han et al., 2007). Cinobufagin, a hydrophobic compound, shows a great ability to inhibit the tumor cell by a pilot study and is allowed to be used for injection in a clinic that is approved by the Chinese Food and Drug Administration (CFDA) (Meng et al., 2009). Bufadienolides is mainly extracted from medicinal toads but with a high economic cost. In addition, the medical resources of bufadienolides are in short supply. Recently, synthetic biology has played an increasingly important role in valuable natural products.

At present, the biosynthesis of bufadienolides is complex and unclear. Depending on the isotope labeling results, cholesterol is the starting compound and bile acid is the important intermediate involved in endogenous bufadienolides biosynthesis of mammals, and *Bufo* was researched in the 1970s (Porto and Gros, 1970, 1971; Porto et al., 1972). Recently, two important enzymes, 3*β*-hydroxysteroid dehydrogenase (3*β-*HSD) and steroid 5*β*-reductase (5*β-*POR), were characterized from *Bufo* species and are possibly involved in the bufadienolides biosynthetic pathway. 3*β-*HSD, which belongs to the short-chain dehydrogenase/reductase (SDR), can catalyze two sequential reactions to generate progesterone, including converting the 3β-hydroxysteroid to 3-oxo intermediate and then isomerizing the Δ^5^ form to the Δ^4^ form and the enzyme is investigated to be accumulated nearly 20-fold in venom glands than in liver (Xu et al., 2018). Then, the 5*β-*POR encodes the progesterone 5*β*-reductase, which is also confirmed *in vitro* (Zhang et al., 2021). The biosynthetic pathway of bufadienolides is so complicated that the relating key enzymes still need to be deeply illustrated by the method of transcriptomics.

Another essential enzyme, P450scc, was investigated to be possibly involved in the cardiac glycosides biosynthesis pathway in plants such as Scilla and Urginea (Sauer et al., 1967; Stache et al., 1969), *Calotropis procera* (Pandey et al., 2016). It is interesting to explore the correlation of biosynthetic pathways and bufadienolides compounds between the animal and plant sources. It is noteworthy that abnormal steroidogenesis could cause serious disease in the human body (Zhang et al., 2012). The demand for steroid precursors seems to continuously enlarge due to the increasing usage of steroid drugs, such as glucocorticoids, mineralocorticoids, and sex hormones. According to a biosynthesis study of steroid hormone compounds in animals, P450scc, as a key enzyme, can catalyze cholesterol to produce the biologically active compound pregnenolone (Hochberg et al., 1974; Duque et al., 1978; Efimova et al., 2019), which plays an important role in all classes of steroidal drug biosynthetic pathways. This reaction first contains two hydroxylation steps on the side chain of cholesterol at the C22 and C20 positions to produce 22R-hydroxycholesterol and 20α, 22R-dihydroxycholesterol, and then scission of the side chain between C20-C22 (Strushkevich et al., 2011). The identification of BbgP450scc enriched the species diversity of this enzyme and was of significance to study the characteristics of BbgCYP11A1, such as substrate specificity, enzyme kinetics.

In addition, a complete transcriptomic analysis of different tissues (skins, venom glands, and muscles) from the *Bufo* species is needed. In this study, we systematically investigated the transcriptome analysis of *B. bufo gargarizans* in three different tissues to understand the molecular basis of bufadienolides biosynthesis. Through the transcriptome sequencing of *B. bufo gargarizans*, candidate genes of the P450scc system were isolated with a homologous gene from *G. rugose*, *X. tropicalis*, *X. laevis*, and *N. parkeri* as probes. The biochemical characterization of the BbgP450scc system successfully catalyzed cholesterol into pregnenolone by heterologous expression in an engineering yeast producing cholesterol. These data provide a comprehensive resource to speculate on the biosynthesis of *B. bufo gargarizans*.

## Materials and Methods

### Animal Materials and RNA Extraction

Three *B. bufo gargarizans* were obtained from Foshan, Guangdong Province in China. Animal treatment and experimental protocols were conducted in accordance with the Animal Care Guidelines of Guangzhou University of Chinese Medicine. After being sacrificed, three tissues, including muscles, skins (containing glands), and venom glands from three toads, were collected, respectively. All samples were frozen immediately in liquid nitrogen and then stored in −80°C for further processing. Total RNA was extracted using a Quick RNA Isolation Kit (Huayueyang Biotechnology) according to the manufacturer’s protocol. The quality and quantity of total RNA were assessed using 1% agarose gels and a Qubit 2.0 Fluorometer (Invitrogen, United States).

### cDNA Library Construction for Sequencing and *de novo* Assembly

The mRNA was purified from the total RNA using mRNA capture beads and subsequently sheared short fragments under the condition of divalent cations and high temperatures. Further, these fragments were reverse-transcribed into first-strand cDNA by random hexamers. Then, second-strand cDNA was synthesized in the presence of DNA Polymerase I and RNase H. Clean cDNA was produced using 1 × Ampure XP DNA clean beads after a series of operations, including end repair, addition of “A” base and ligation of Illumina adapters. For the enrichment of adapter ligated fragments, 15 cycles of PCR reaction were carried out to amplify cDNA libraries. Nine cDNA libraries were sequenced on the Illumina Hiseq Nova Sequencing platform, and 150 bp paired-end reads were generated. Quality control of raw reads was performed by FastQC. Reads with unknown nucleotides and low-quality reads less than 20 bp were trimmed using Prinseq software (Bolger et al., 2014) and sequence contamination was assessed by blast+ in NCBI (Altschul et al., 1997). Clean reads were assembled into the unigenes by Trinity software v2.4.0 (Haas et al., 2013).

### Sequence Annotation and Functional Classification

Functional annotation was performed by BLASTP against the protein databases NR (NCBI nonredundant proteins sequences), PFAM (Protein family), CDD (Conserved Domain Database), KOG (eukaryotic Ortholog Groups), GO (Gene Ontology), KEGG (Kyoto Encyclopedia of Genes and Genomes), Swiss-Prot (a manually annotates and reviewed protein sequence database), TrEMBL, and NT (NCBI nucleotide sequences). GO terms were mainly divided into three parts, including biological processes, molecular functions, and cellular components. GO enrichment analysis was implemented by the BLAST result from the UniProt database. The *B. bufo gargarizans* gene set was also mapped to the KEGG pathway database to identify the best match for each gene (Gerlich and Neumann, 2000).

### Quantification of the Differentially Expressed Gene Expression Levels

Gene expression levels were calculated using Salmon (Patro et al., 2017), and the read count for each gene in various organs was normalized into a transcripts per million (TPM) value. Genes with TPM value >3 were selected for further differentially expressed gene (DEG) analysis. DEGs among muscles, skins, and venom glands, were performed by DESeq software. The unigenes with a |Fold Change| > 2 and an adjusted *p*-value (*q-*value) < .05 were considered as DEGs. GO enrichment analysis of DEGs was carried out by topGO, and KEGG enrichment analysis of DEGs was implemented by clusterProfiler (Yu et al., 2012). DEGs from GO terms and KEGG terms with *q*-value< 0.05 were considered to be considerably enriched.

### Phylogenetic Analyses and Multiple Alignment of P450scc System

To identify the nucleotide and deduced amino acid sequences of the cloned cDNA sequence, a neighbor-joining tree was generated using MEGA7 software with default parameters based on 1,000 bootstrap replications (Sudhir et al., 2016). Evolview (https://www.evolgenius.info/evolview/) was utilized to edit the phylogenetic trees. Multiple sequence alignment was implemented by DNAMAN software (version 6.0).

### Validation of P450scc System by Quantitative Real-Time PCR

Total RNA of nine samples from three tissues were isolated by a Quick RNA Isolation Kit (Huayueyang Biotechnology), and the cDNA was synthesized using the Evo M-MLV One Step RT-PCR Kit (Accurate Biotechnology). The sequence encoding BbgP450scc system, including BbgCYP11A1, BbgAdx (adrenodoxin) and BbgAdR (adrenodoxin reductase), was obtained from the transcriptome data. Here, the housekeeping gene *β-actin* from *B. bufo gargarizans* was used as an internal reference gene (Wu et al., 2017). Real-time PCR reactions were performed using SYBR ® Green Premix ProTaq HS qPCR Kit (Accurate, Hunan, China) on the QuantStudio^TM^ 5 Real-Time PCR Instrument (384-Well Block) (Thermo Fisher Scientific, United States). Each reaction was performed in a final volume of 10 μL, containing 8.2 μL SYBR Green PCR Master Mix, 10 mM of each primer, and 1 μL cDNA template. The amplification program was as follows: 50°C for 20 s, 90°C for 30 s, followed by 40 cycles of 95°C for 15 s and 60°C for 34 s. All reactions were performed with three biological replicates. The relative expression level of genes was calculated by the 2^−ΔΔCt^ method (Livak and Schmittgen, 2002). Primers used in the qRT-PCR detection were listed in Supplementary Table S4.

### Cloning of P450scc System Genes

The cDNA sample of *B. bufo gargarizans* was used for gene cloning. The full-length cDNA sequences of BbgAdx (MZ 384243) and BbgAdR (MZ 384244) were obtained by PCR reaction. The nucleotide sequences of redox partners from *Mus musculus* were downloaded from NCBI database and named as MmAdx (NM 007996.2) and MmAdR (NM 007997.1). A 2A peptide linker was used to fuse the redox partners (Ryan and Drew, 1994). The fused proteins were derived by overlapping PCR and named as CTL-1 (BbgAdx-2A-BbgAdR) and CTL-2 (MmAdx-2A-MmAdR). Both of them were cloned into the pESC-Leu vector (Agilent Technologies, Inc.) to obtain plasmids pGL01 and pGL02 using the ClonExpress^®^ II One Step Cloning Kit (Vazyme, Nanjing, China).

The full-length cDNA sequence of BbgCYP11A1 (MZ 384245) was amplified by using RACE PCR methods and then cloned into plasmids pESC-Leu, pESC-Leu-AaCPR (constructed previously from our laboratory), pGL01 and pGL02, respectively, to obtain vector pGL03, pGL04, pGL05 and pGL06. The primers and sequences used in this section are shown in Supplementary Table S5 and Supplementary Table S6, and detailed information about the plasmids is listed in Supplementary Table S7.

### Heterologous Expression of P450scc System in Saccharomyces cerevisiae

A cholesterol producing strain, RH6829 (*MAT*a *ura3 leu2 his3 trp1 can1 bar1 erg5Δ:: HIS5-TDH3-DHCR24 erg6Δ:: TRP1-TDH3-DHCR7*), was kindly gifted by Professor Howard Riezman (Souza et al., 2011). The expression plasmids, pGL03, pGL04, pGL05, and pGL06, were transformed into these strains to get strains GL01, GL02, GL03, and GL04, respectively. The transformants were selected on SD-Trp-His-Leu medium containing 2% glucose and further identified by the PCR method. Positive colonies of these strains were cultured in 50 ml SD-Trp-His-Leu medium with 18% galactose and 2% glucose for 7 days with shaking at 220 rpm and 30°C.

### Extraction and GC-MS Analysis of Steroid Compounds

The cultures of each strain were centrifuged at 4000 rpm for 5 min, and the cells were harvested and then lysed in 10 ml saponification reagent [20% (w/v) KOH in 50% (v/v) ethanol]. After being extracted twice by equal volumes of *n*-hexane, the organic phase extracts were collected and dried by a centrifugal vacuum evaporator. The crude extract was derived by using 30 μL N-Methyl-N-(trimethylsilyl) trifluoroacetamide (Sigma-Aldrich, China). Then, the reactant was analyzed using a Shimadzu QP2010SE instrument under the electronic impact of 70 eV with a Rxi-5Sil MS capillary column (0.25 mm × 30 m) (Shimadzu, China). The sample analysis on Gas chromatography mass spectrometry (GS-MS) was performed according to the previous report (Zhang et al., 2019).

## Results

### Transcriptome Sequencing and *de novo* Assembly

**FIGURE 1:**
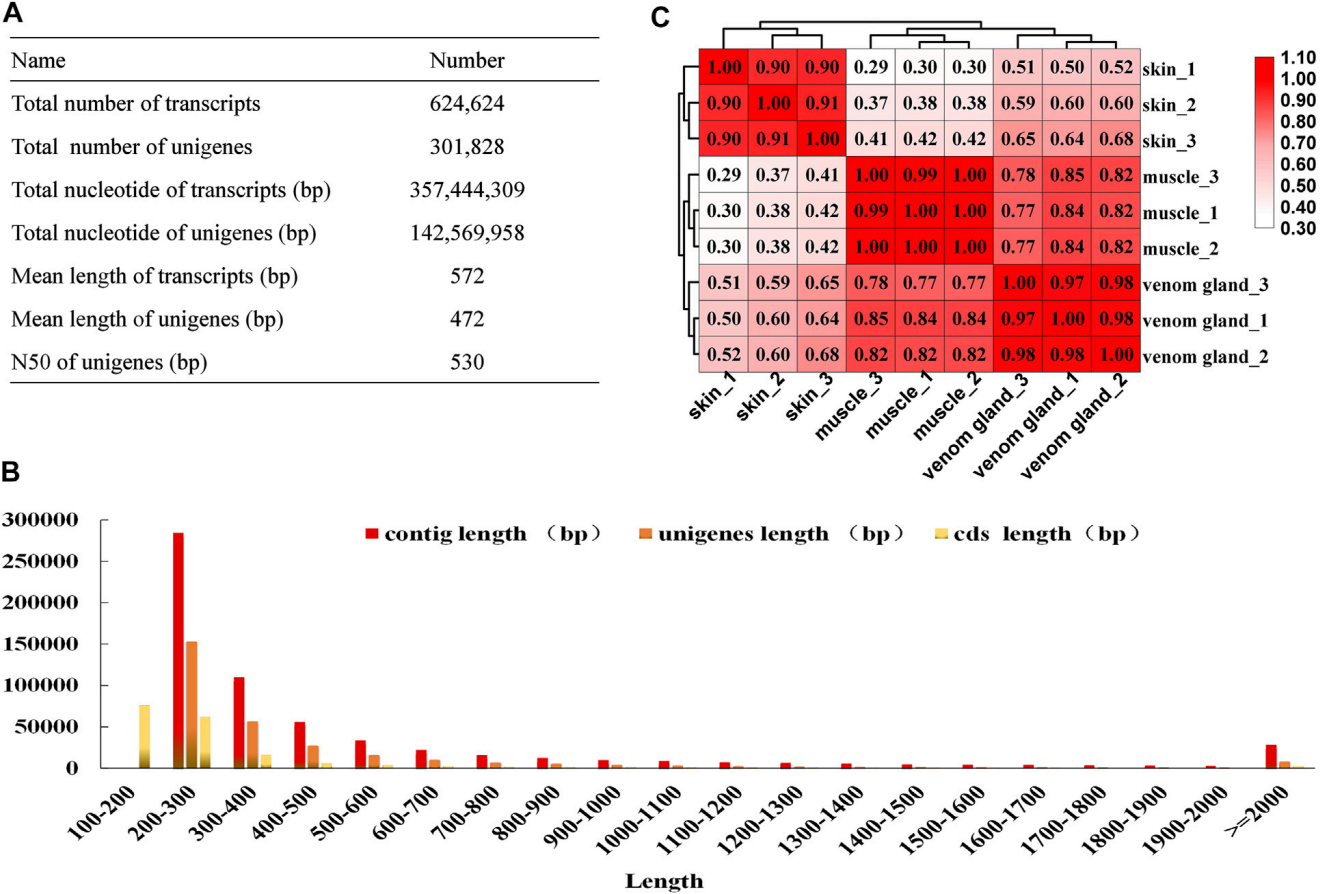
Illumina sequencing of different tissues of *B. bufo gargarizans*. **(A)** The results of the assembly sequences. **(B)** Pearson’s correlation coefficients of the biological replicates among three tissues. **(C)** The length distribution of contigs, unigenes, and CDSs.

To obtain the key genes of the bufadienolides biosynthetic pathway, RNA-seq was applied using three tissues (muscles, skins, and venom glands) based on the bufadienolides accumulation pattern in *B. bufo gargarizans*. Nine cDNA libraries were constructed and sequenced on the Illumina Hiseq Nova Sequencing platform with a pair-end length of 150 bp. Approximately 493.8 million raw reads were obtained, and after filtering low-quality reads, adaptor, and short reads, 443.9 million clean reads were generated with the Q30 > 96% (Supplementary Table S1). All the clean reads were *de novo* assembled into 624,624 transcripts, and after redundancy elimination, 301,828 unigenes were identified with a mean length of 472 bp and an N50 length of 530 bp (Figure 1A). The length distribution of transcripts, unigenes, and CDSs is presented in Figure 1B. For unigenes, 2.56% of the unigenes were >2000 bp in length, and most sequence lengths were between 200 and 600 bp. Pearson’s correlation coefficients of the biological replicates of each tissue were more than 0.90, indicating their high correlation (Figure 1C). These results reveal that the sequencing data has high quality and can be used in the subsequent analysis.

### Functional Annotation and Classification of Unigenes

**FIGURE 2:**
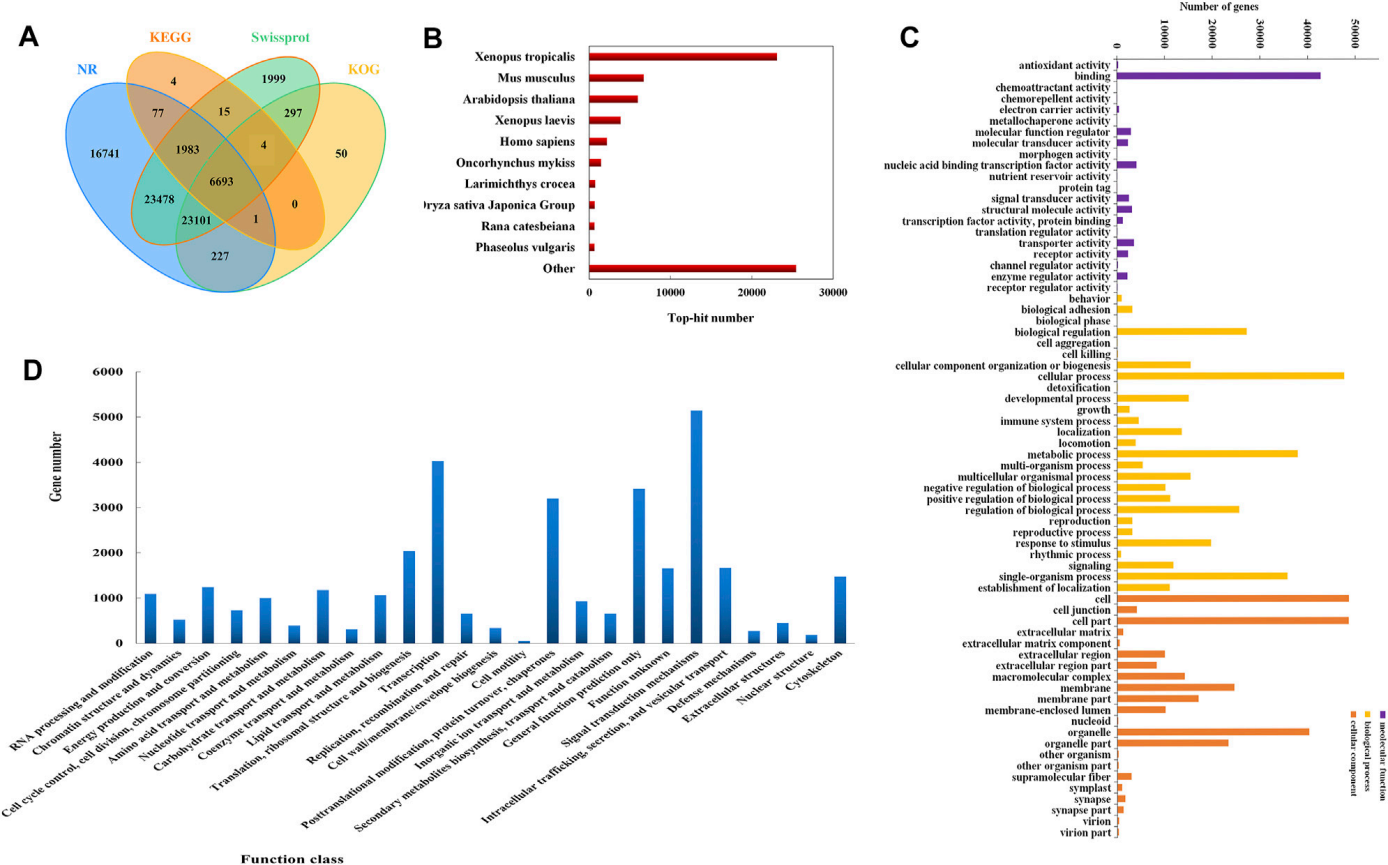
Results of unigenes classification. **(A)** The annotation number of unigenes in NR, KEGG, Swissprot, KOG databases. **(B)** Distribution of annotated unigenes in homologous species by searching NR database. **(C)** Annotation of GO classification of unigenes in the transcriptome. GO classification included three main categories: molecular function, biological process, and cellular component. **(D)** KOG annotation of the unigenes.

Total 301,828 unigenes were annotated against nine publicly available databases comprising Conserved Domain Database (CDD), Kyoto Encyclopedia of Genes and Genomes (KEGG), NCBI non-redundant protein sequences (NR), NCBI nucleotide sequences (NT), Protein family (PFAM), a manually annotates and reviewed protein sequence database (Swiss-Prot), TrEMBL, Gene Ontology (GO) and eukaryotic Ortholog Groups (KOG), and 86,783 unigenes had significant hits in at least one database (Figure 2A, Supplementary Table S2). Most unigenes were annotated in the NR database (23.95%), and the annotated sequences were blasted against other species. The result of species distribution showed 23,096 homologous unigenes between *B. bufo gargarizans* and *Xenopus tropicalls*, indicating their high homology (Figure 2B)*.*

A total of 652,248 unigenes were classified into three main GO functional categories, corresponding to molecular function (93,600), biological process (325,157), and cellular components (258,683). The three categories were further divided into 21, 27, and 22 terms, respectively. The majority of unigenes in the biological process belong to the cellular process (47,642), then followed by the metabolic process (37,904). In the category of molecular function, ATP binding was the main term, which was of 42,741 unigenes. In addition, most unigenes in the cellular component were assigned to cell (48,584) and cell part (48,537) (Figure 2C).

33,696 unigenes were assigned into 25 different groups in KOG analysis, and the largest group was “signal transduction mechanisms”, which consists of 5141 members. Moreover, there were 657 unigenes clustered into “secondary metabolites biosynthesis, transport, and catabolism”, and the unigenes between them may be involved in the bufadienolides biosynthetic pathway (Figure 2D).

### Differential Expression Genes Among Tissues

**FIGURE 3:**
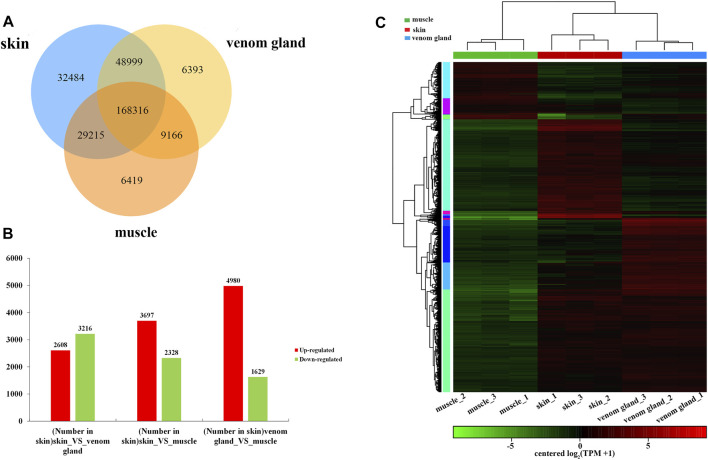
Analysis of DEGs in the transcriptome data. **(A)** Venn diagram represents the number of differential expression genes among three tissues: skin, venom gland, and muscle. **(B)** The number of upregulated and downregulated genes of in each tissue compared with two other tissues. **(C)** The clustering heat map of differential gene expression.

To investigate the DEGs, the DEGs were identified based on the values of TPM among different tissues. The figure showed 168,316 genes expressed in all three tissues, whereas there were 32,484 genes specifically expressed in the skins, followed by 6419 in muscles and 6393 in the venom glands (Figure 3A). Compared with the muscles, there are 3697 upregulated genes and 2328 downregulated genes were identified in the skins, whereas in the venom glands, 4980 upregulated genes and 1629 downregulated genes were detected (Figure 3B). Compared with the venom glands, there were 2608 upregulated and 3216 downregulated genes in the skins (Figure 3B, Supplementary Sheet S1). The expression patterns of all DEGs among three tissues were hierarchically clustered in the heat map (Figure 3C). The DEGs of venom glands were clustered together with the DEGs of skins in a subclade. However, genes in the muscles were clustered into a different clade that indicates their significant discrepancy.

KEGG enrichment analysis was performed to explore the potential DEGs in different metabolism pathways. Compared with the muscles, there were 1485 upregulated genes in the skin and 1694 upregulated genes in the venom glands. These DEGs were enriched in several pathways, including terpenoid backbone, steroid, steroid hormone, and primary bile acid biosynthesis (Supplementary Figure S1, Supplementary Figure S2 and Supplementary Figure S3).

### Candidate Genes Involved in Putative Bufadienolides Biosynthesis

**FIGURE 4:**
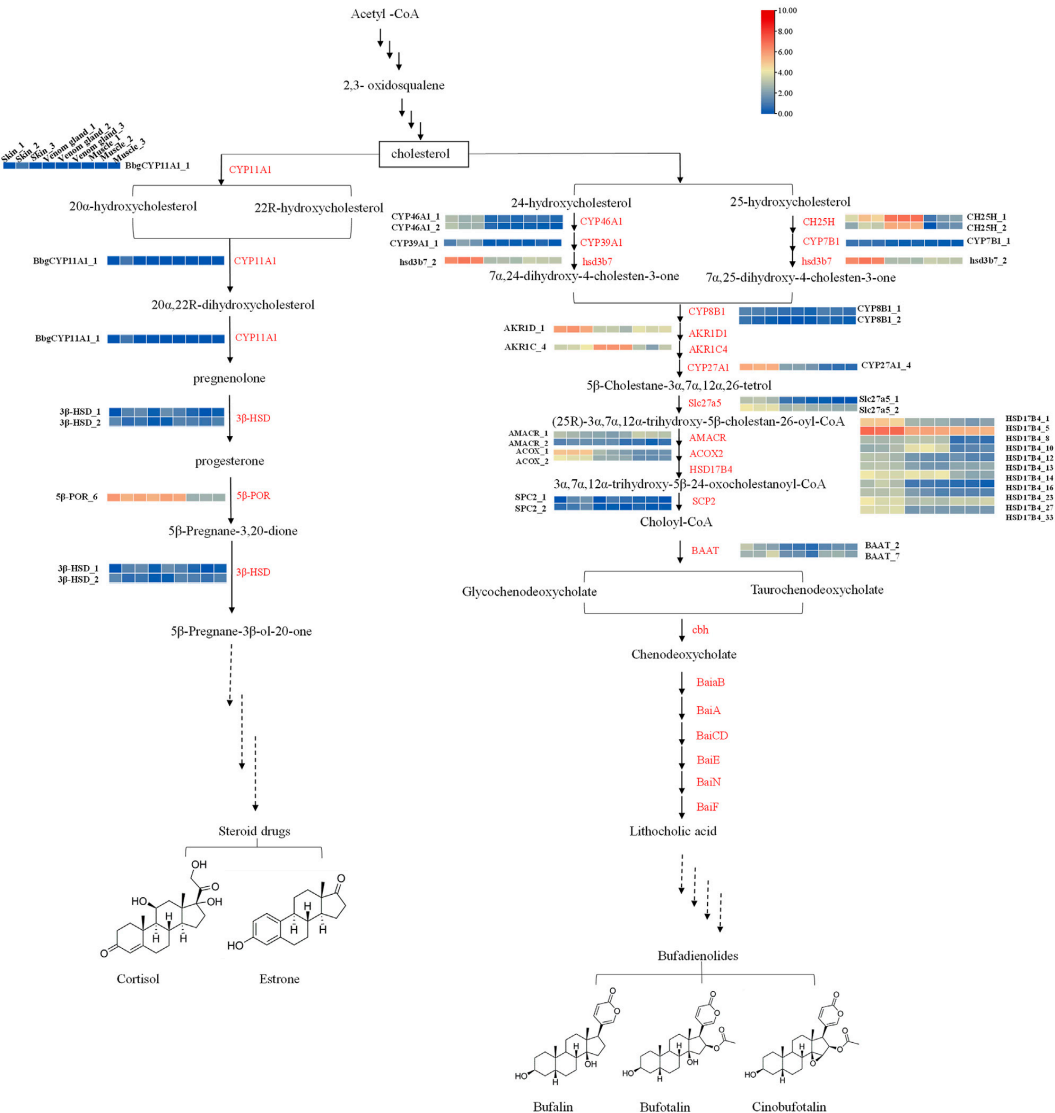
The enzymes are possibly involved in pregnenolone and the bufadienolides biosynthesis pathway (Payne and Hales, 2004; Norlin and Wikvall, 2007). Heat maps display the differential expression of transcripts encoding enzymes involved in different steps. In the heat maps, different columns represent tissues in order of skin, venom gland, and muscle from left to right. Color scale representing normalized expression values is shown at the top. The red names in the pathways correspond to Supplementary Table S3.

**FIGURE 5:**
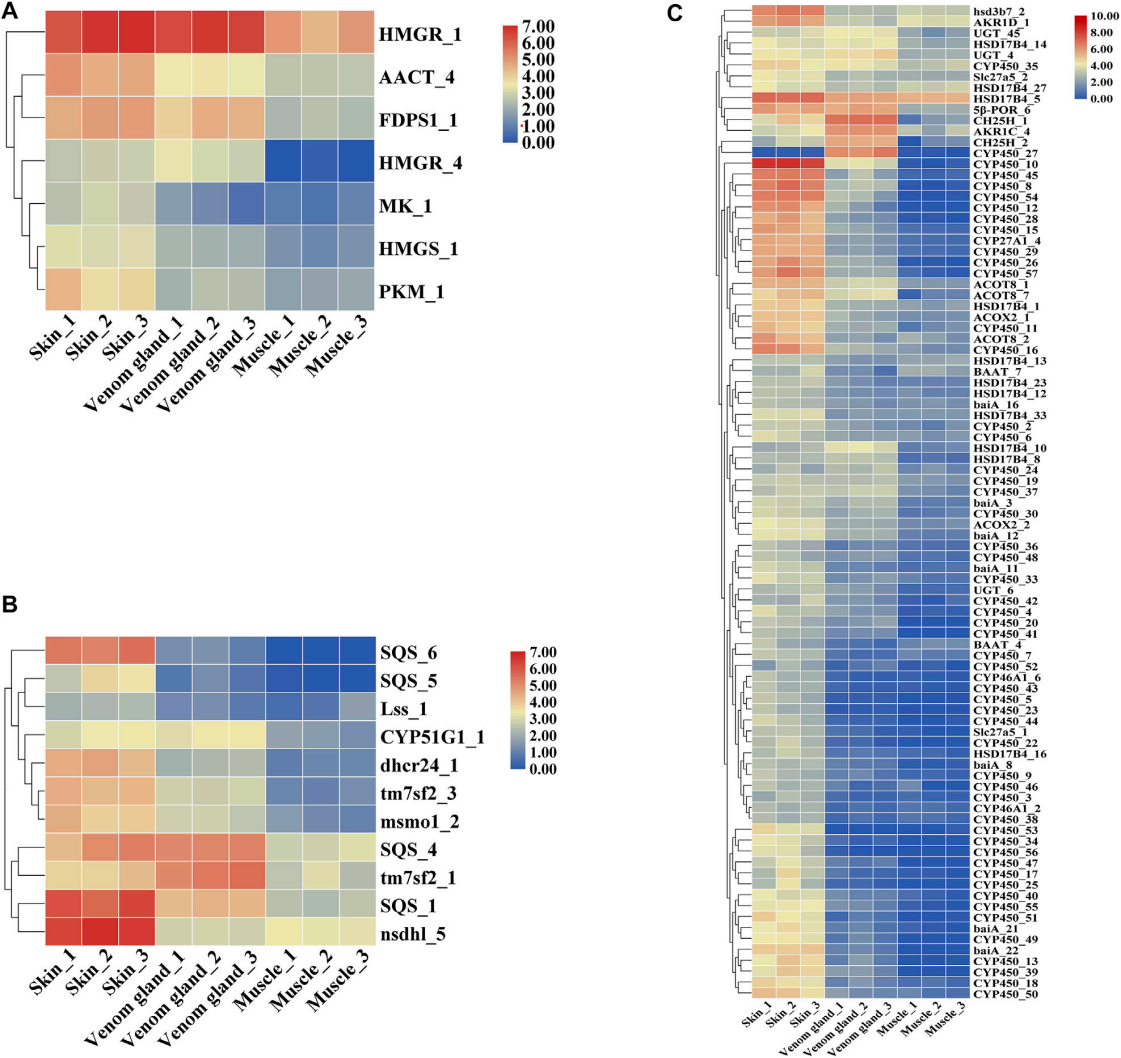
The heat map of the expression pattern of genes involved in pregnenolone and bufadienolides biosynthetic pathway. **(A)** Upstream terpenoid backbone biosynthetic pathway, **(B)** Midstream cholesterol biosynthetic pathway, **(C)** Downstream pregnenolone and bufadienolides biosynthetic pathway.

Candidate genes were screened via homology-based searches of the transcriptome. In total, 389 unigenes were predicted to be involved in the bufadienolides metabolic pathway, which can be divided into three parts: upstream terpenoid backbone biosynthetic, midstream cholesterol biosynthetic, and downstream bufadienolides biosynthetic pathways (Figure 4, Supplementary Figure S4). In terpenoid backbone biosynthesis, 42 candidate genes encoding seven enzymes were identified as follows: AACT, Acetyl-CoA C-acetyltransferase (16 unigenes); HMGS, hydroxymethylglutaryl-CoA synthase (4 unigenes); HMGR, hydroxymethylglutaryl-CoA reductase (NADPH) (13 unigenes); MK, mevalonate kinase (2 unigenes); PKM, phosphomevalonate kinase (2 unigenes); MVD, diphosphomevalonate decarboxylase (1 unigene); FDPS, farnesyl diphosphate synthase (4 unigenes). Through the expression pattern analysis, seven unigenes encoding six enzymes were identified as DEGs in the upstream pathway. According to the heat map as shown, the enzymes AACT_4 and FDPS1_1 showed higher expression in the skin than in the venom glands (Figure 5A, Supplementary Sheet S2).

In the midstream pathway, there were 53 candidate genes encoding 12 enzymes that may involve the cholesterol biosynthesis pathway (e.g., SQS, squalene synthase; SQE, squalene monooxygenase; Lss, lanosterol synthase; tm7sf2, Delta14-sterol reductase; msmo1, methyl sterol monooxygenase, etc*.*). In addition, 11 unigenes encoding seven enzymes were selected as DEGs. The enzyme SQS showed the highest expression in the skins and the lowest expression in muscles. The enzymes dhcr24 and tm7sf2 also have the similar expression patterns with the SQS (Figure 5B, Supplementary Sheet S2).

The downstream pathway may relate to the bile acid biosynthesis pathway in animals (Fedorova et al., 2015). Bile acid is a common production in the liver in animals (Reuben, 1987; Russell and Setchell, 1992). The enzymes related in the metabolic pathway of Figure 4 were predicted according to bile acid and steroid hormone biosynthesis from the KEGG pathway and those previous studied (Payne and Hales 2004; Norlin and Wikvall 2007). There were 145 unigenes encoding 19 enzymes (CYP46A1, CYP39A1, HSD3B7, CH25H, etc*.*) that may relate to the bile acid biosynthesis pathway (Figure 4). For the downstream pathway, 294 candidate genes encoding 27 enzymes, including 52 unigenes of cytochromes P450 that may involve the pregnenolone and bufadienolides biosynthesis pathways, were predicted. The heat map showed that most of DEGs had relatively high expression in the skin (Chen et al., 2020) (Figure 5C, Supplementary Sheet S2).

### Phylogenetic Analyses and Multiple Alignment of BbgP450scc System

**FIGURE 6:**
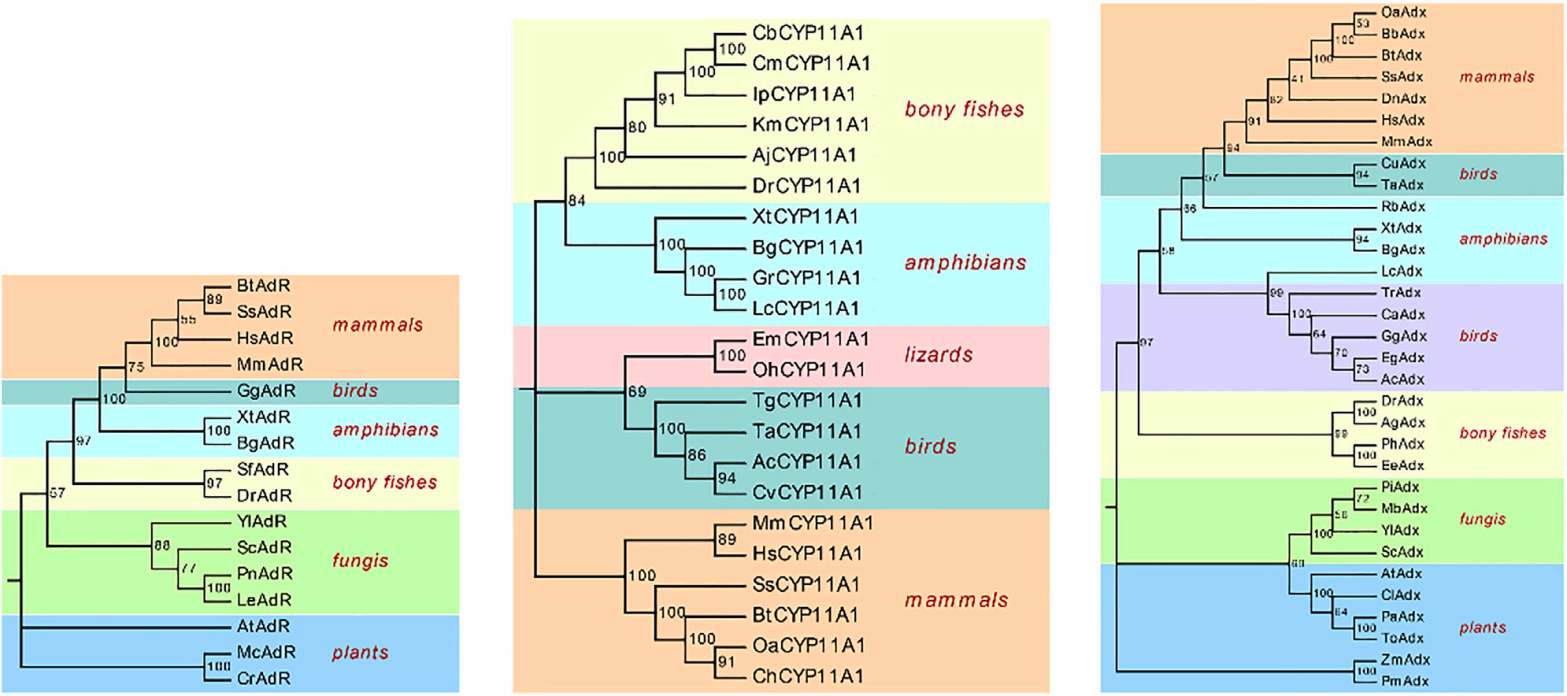
Phylogenetic analysis of P450scc system. The reference sequences of CYP11A1, Adx, AdR from different species were selected from NCBI, including mammals, amphibians, birds, bony fish, plants, fungi, lizards. Xt, *Xenopus tropicalis*; Ta, *Tyto alba*; Aj, *Anguilla japonica*; Km, *Kryptolebias marmoratus*; Gr, *Glandirana rugose*; Bt, *Bos taurus*; Gg, *Gallus gallus*; Dr, *Danio rerio*; Mm, *Mus musculus*; Hs, *Homo sapiens*; Lc, *Lithobates catesbeianus*; Ss, *Sus scrofa*;Oa, *Ovis ammon*; Ch, *Capra hircus*; Cb, *Clarias batrachus*; Cm, *Clarias magur*; Ip, *Ictalurus punctatus*; Ac, *Anser cygnoides*; Tg, *Taeniopygia guttata*; Cv, *Colinus virginianus*; Oh, *Ophiophagus Hannah*; Em, *Eublepharis macularius*; Dn, *Dasypus novemcinctus*; Bb, *Bubalus bubalis*; Lr, *Labeo rohita*; Ag, *Anabarilius grahami*; Ph, *Pangasianodon hypophthalmus*; Ee, *Electrophorus electricus*; Eg, *Egretta garzetta*; Tr, *Turdus rufiventris*; Ca, *Calypte anna*; Ac, *Antrostomus carolinensis*; Cu, *Catharus ustulatus*; Rb, *Rhinatrema bivittatum*; Yl, *Yarrowia lipolytica*; Sc, *Saccharomyces cerevisiae*; Pi, *Penicillium italicum*; Mb, *Metarhizium brunneum*; Pa, *Parasponia andersonii*; To, *Trema orientale*; Cl, *Carex littledalei*; Zm, *Zea mays*; Pm, *Panicum miliaceum*; At, *Arabidopsis thaliana*; Sf, *Salvelinus fontinalis*; Mc, *Micractinium conductrix*; Cr, *Chlamydomonas reinhardtii*; Pn, *Pyrrhoderma noxium*; Le, *Lentinula edodes*; Yl, *Yarrowia lipolytica*; Bn, *Bugula neritina*; Bbg, *Bufo bufo gargarizans*.

To employ the relationship of BbgP450scc and the biosynthesis of bufadienolides, BbgP450scc gene was screened from transcriptome data. P450scc is also an important first step enzyme participating in steroid drugs biosynthesis and can convert cholesterol to pregnenolone, which is the key precursor to synthesize all classes of steroidal drugs. Two short sequences TRINITY_DN118184_c0_g1 (BbgCYP11A1_1) and TRINITY_DN130445_c0_g1 (BbgCYP11A1_2) were screened from the transcriptome data by BLASTP. BbgCYP11A1_1, and BbgCYP11A1_2 encoded 73 and 49 amino acids, respectively. Only the sequence of BbgCYP11A1_1 could be derived by RACE-PCR for the subsequent research. The complete sequence BbgCYP11A1_1 encoding 512 amino acids was isolated from the cDNA library. At the same time, two unigenes (BbgAdR_1, BbgAdR_2) were annotated as adrenodoxin oxidoreductase and six unigenes (BbgAdx_1, BbgAdx_2, BbgAdx_3, BbgAdx_4, BbgAdx_5, BbgAdx_6) were annotated as adrenodoxin. Full ORF sequences are found in BbgAdx_1 and BbgAdR_1, encoding 167 and 494 amino acids, respectively. A phylogenetic tree was constructed using the protein sequences with CYP11A1, Adx, and AdR from the species of mammals, birds, amphibians, and bony fish, etc*.* (Figure 6). As the multiple sequence alignment result shows, the deduced amino acid sequences BbgCYP11A1 had more than 78% identity with Amphibian CYP11A1 (Supplementary Figure S5).

### Isolation and Tissue Expression Profile of BbgP450scc System Genes

**FIGURE 7:**
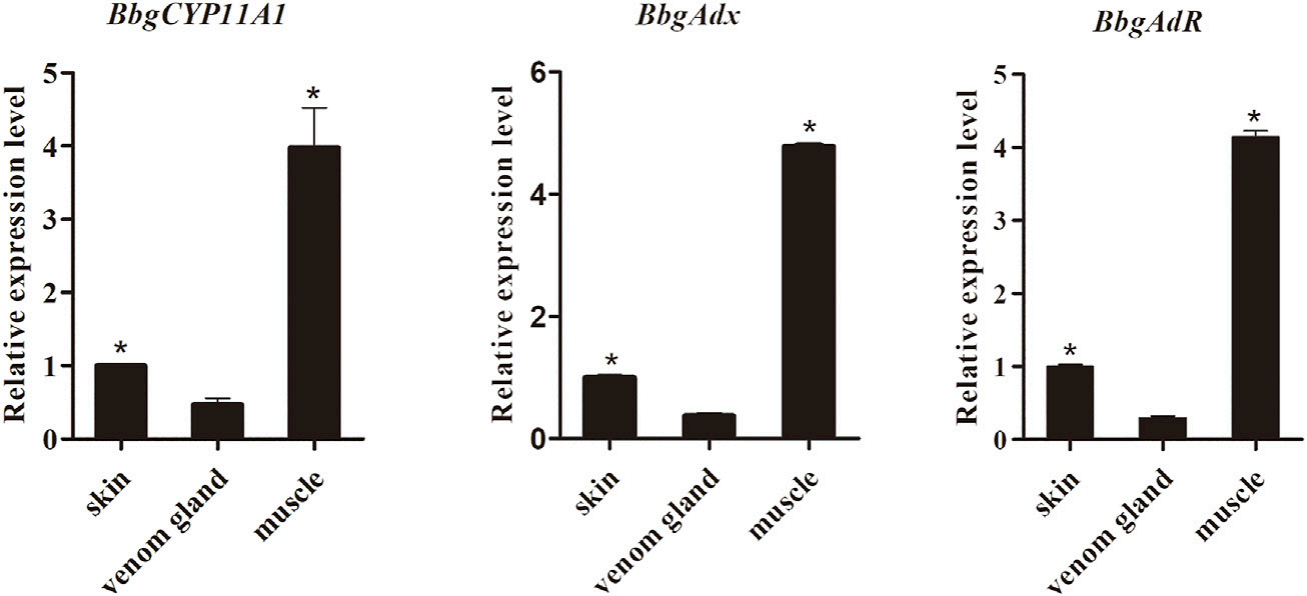
The expression pattern of genes in P450scc system among different tissues of *B. bufo gargarizans*. (*p < .05).

Aimed to verify the biochemical function of BbgP450scc system genes, including BbgCYP11A1 and its redox partners BbgAdx and BbgAdR, the complete sequences of them were acquired and qRT-PCR was carried out to detect the expression patterns of BbgP450scc system genes. BbgP450scc system genes had similar expression profiles showing the higher accumulation in muscles than in venom glands and in skins (Figure 7).

### Functional Characterization of BbgP450scc System in Yeast

**FIGURE 8:**
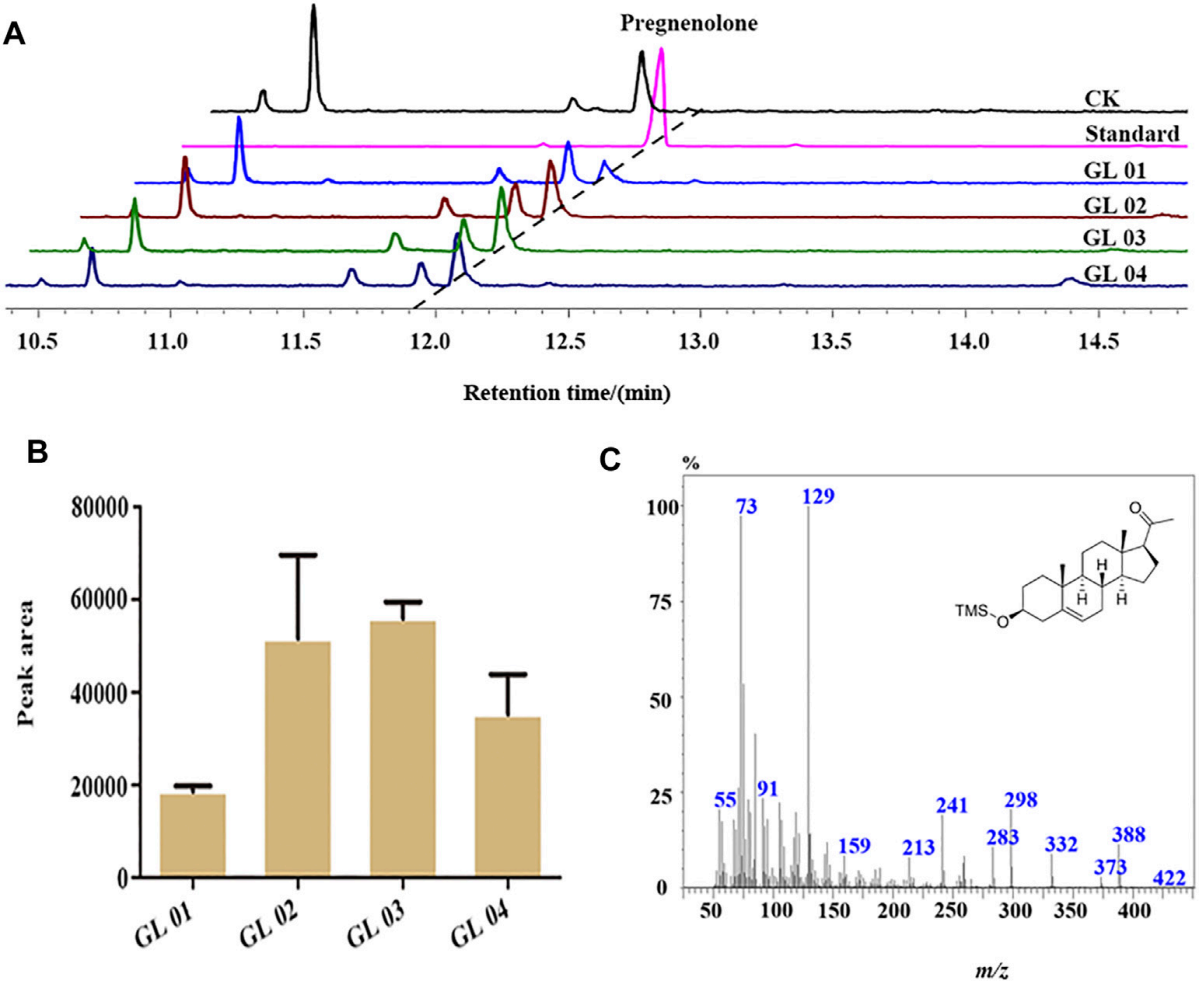
GC-MS analysis of the products in the cultures of BbgCYP11A1 transformed yeast. **(A)** GC-MS total ion chromatograms examination the products of BbgCYP11A1 with various redox partners in yeast. CK, blank plasmid in strain; Standard, the compound of pregnenolone; GL01, Plasmid pESC-Leu-BbgCYP11A1 in strain; GL02, Plasmid pESC-Leu-BbgCYP11A1-AaCPR in strain; GL03, Plasmid pESC-Leu-BbgCYP11A1-BbgAdx-2A-BbgAdR in strain; GL04, Plasmid pESC-Leu-BbgCYP11A1-MmAdx-2A-MmAdR in strain. **(B)** The peak area of BbgCYP11A1 for pregnenolone production with different redox partners. **(C)** The mass spectrum of the chemical in the samples of transforming yeast at 11.95 min of retention time.

P450scc can convert cholesterol, campesterol, 7-reductase ergosterol, β-sitosterol, and lumisterol into pregnenolone. As the best substrate, cholesterol can be fully converted to pregnenolone by P450scc (Tuckey et al., 2014; Gerber et al., 2015). To characterize the catalytic function of BbgCYP11A1, the heterologous vector pGL05 containing BbgCYP11A1 was transformed into the cholesterol-producing strain RH6829 (Souza et al., 2011). The catalytic product was identified as pregnenolone by comparing the MS spectrum and retention time with the standard (Figure 8A and Figure 8C). In addition, the catalytic efficiency of P450scc is influenced by the redox partners, adrenodoxin reductase (AdR), and adrenodoxin (Adx) (McLean et al., 2005). BbgAdx-2A-BbgAdR (CTL1) from *B. bufo gargarizans*, MmAdx-2A-MmAdR (CTL2) from *M. musculus*, and AaCPR from plant *Anemarrhena asphodeloides* Bunge were employed here to evaluate the catalytic capability of BbgCYP11A1 in yeast. Results show that all the redox partners can work with BbgCYP11A1, and the strain GL02 and GL03 displayed higher production than GL01 and GL04, showing the great function in yeast (Figure 8B).

## Discussion

Cardiac glycosides widely exist in the plant kingdom and in animals (Hollman, 1985). Especially in *Bufo* species, their body is capable of producing bufadienolides, which have similar pharmacologic actions with cardenolides generating in plants. The putative biosynthetic pathway leading to the cardenolides is basically deduced from the radiolabeled precursors. The cholesterol side chain cleaving enzyme is important for the formation of cardenolides, which is still unknown in plants (Sauer et al., 1967). Here, we characterized the function of cholesterol side chain cleaving enzyme in *B. bufo gargarizans*, and the expression level analysis of BbgP450scc system was done by RT-PCR. Our study provides more data for deciphering the process of biosynthesis pathway of bufadienolides in *B. bufo gargarizans*.

As the biosynthesis pathway of bufadienolides in *B. bufo gargarizans* has not been clarified yet, to explore the candidate genes of the bufadienolides biosynthetic pathway is needed. According to the result of the transcriptome sequencing and assembly, we analyzed the expression patterns of all the transcripts involved in the putative biosynthesis pathway of bufadienolides and steroid hormones. The transcripts relating to the bile acid biosynthesis, such as cholesterol 24-hydroxylase, cholestanetriol 26-monooxygenase, showed their higher expression levels in skin and venom glands than in muscles. Cholesterol is proved as the precursor of bufadienolides, which are a group of polyhydroxy C-24 steroids with a six-membered lactone (apyrone) ring at the C-17 position. The heat map in Figure 8C shows that CYPs (8, 10, 12, 15, 28, 45, and 54) has significant differential expression in skin, which demonstrates these P450s enzymes might participate in the modification of its parent nucleus. These results indicate that these DEGs are probably involved in bufadienolides biosynthesis generating in skins. This work also provides the foundation for the identification of genes in the biosynthesis pathway.

Steroid compounds are one of the most routinely used chemicals in disease treatment with extensive pharmacological effects (Kuhl, 2011). Nowadays, the most typical marketed products of steroid drugs are cortisol, progesterone, and sex hormone for the treatment of anti-inflammatory, anti-allergic, and contraceptive agents. Using the approach of genetic engineering to improve the production of precursor pregnenolone is an important step in steroidogenesis producing (Duport et al., 1998; Szczebara et al., 2003; Zhang et al., 2019). Then, different redox partners were used to explore the enzymatic activities of BbgCYP11A1, and the results demonstrate that the confusion of BbgAdx and BbgAdR exhibited the relatively higher catalytic efficiency compared with the confusion of the MmAdx and MmAdR. A redox partner AaCPR from the plant also enhanced the enzymatic activity of BbgCYP11A1. Surprisingly, the hybrid combination of BbgP450scc with plant redox partner AaCPR led to a comparable increase in the production of pregnenolone than other combinations. This result provides a cross-species combinatorial biosynthesis approach for pregnenolone metabolic engineering. Moreover, the endogenous redox partners in yeast also can interact with BbgCYP11A1, further suggesting the existence of cross-species redox chain for animal cytochrome P450s.

## Abbreviations

Bbg, *Bufo bufo gargarizans* Cantor; BLASP, Basic Local Alignment Search Tool; CPR, NADPH-cytochrome P450 reductase; GO, Gene Ontology; KEGG, Kyoto Encyclopedia of Genes and Genomes; NR, NCBI non-redundant protein sequences; NT, NCBI nucleotide sequences; P450scc, cholesterol side chain cleavage enzyme; RACE, rapid amplification of cDNA ends

## Data Availability

The original contributions presented in the study are included in the article/Supplementary Material, further inquiries can be directed to the corresponding authors.
