# Supplementary material for: Transcriptome Analysis and Identification of the Cholesterol Side Chain Cleavage Enzyme BbgCYP11A1 From Bufo bufo gargarizans
Source: Front Genet. 2022 Apr 5;13:828877. doi: 10.3389/fgene.2022.828877 (PMC9037069; doi:10.3389/fgene.2022.828877)
Supplement: Supplementary file 3 [file DataSheet1.docx]

**Supplementary materials**

**
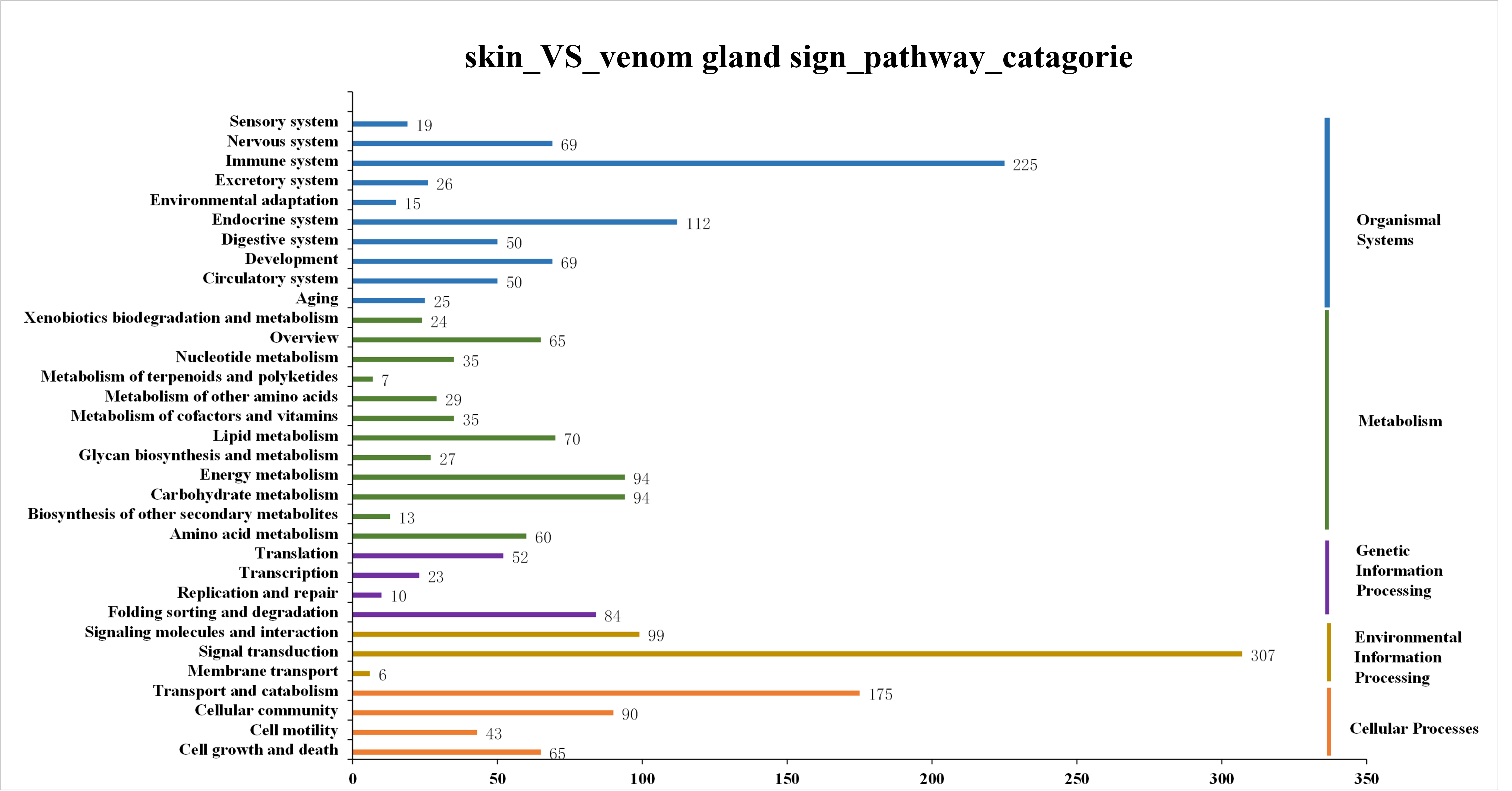
**

**Figure. S1** KEGG annotation of the DEGs in different tissues. Significant enrichment pathways in skin vs. venom gland comparison.

**
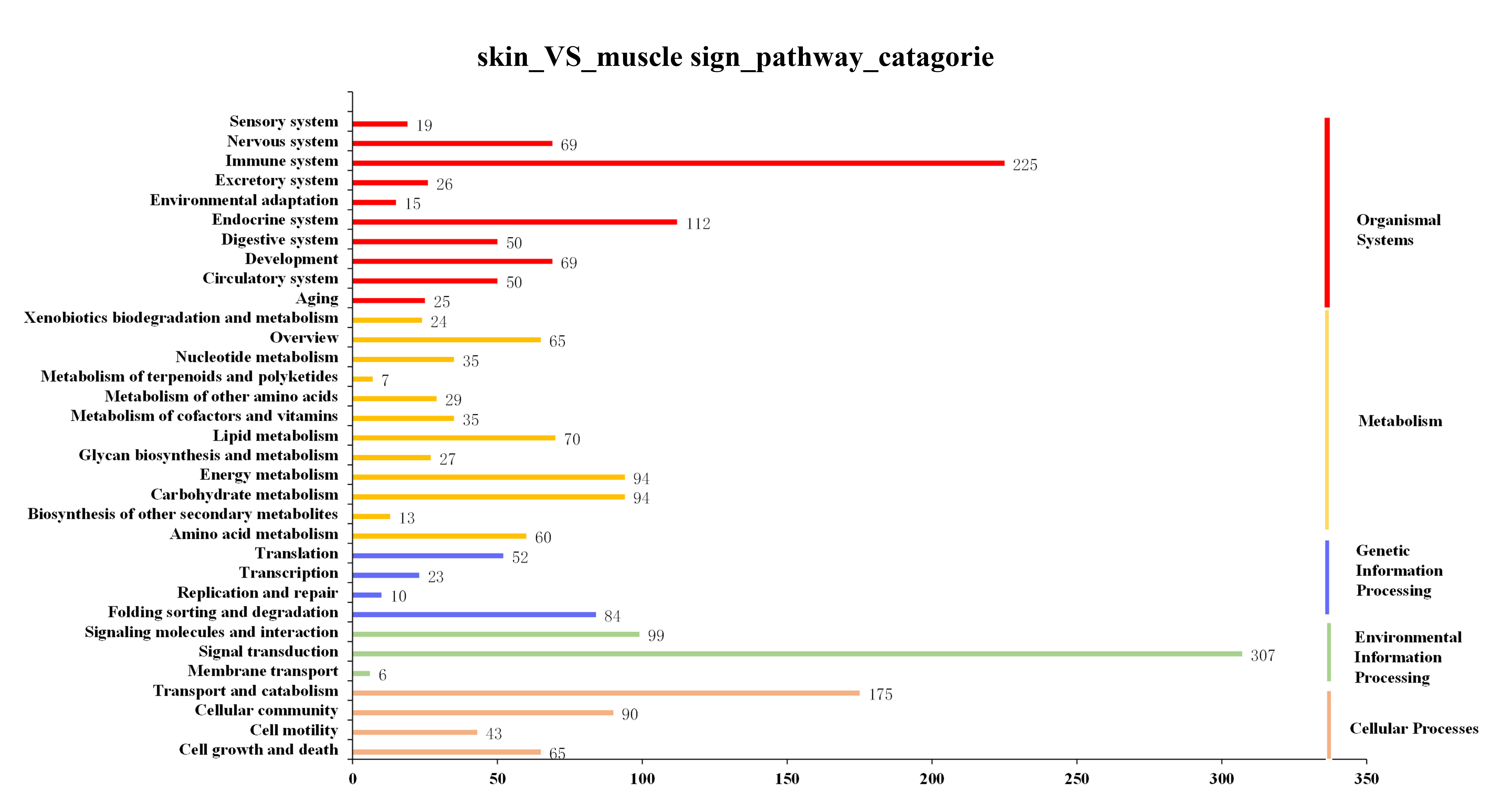
**

**Figure. S2** KEGG annotation of the DEGs in different tissues. Significant enrichment pathways in skin vs. muscle comparison.

**
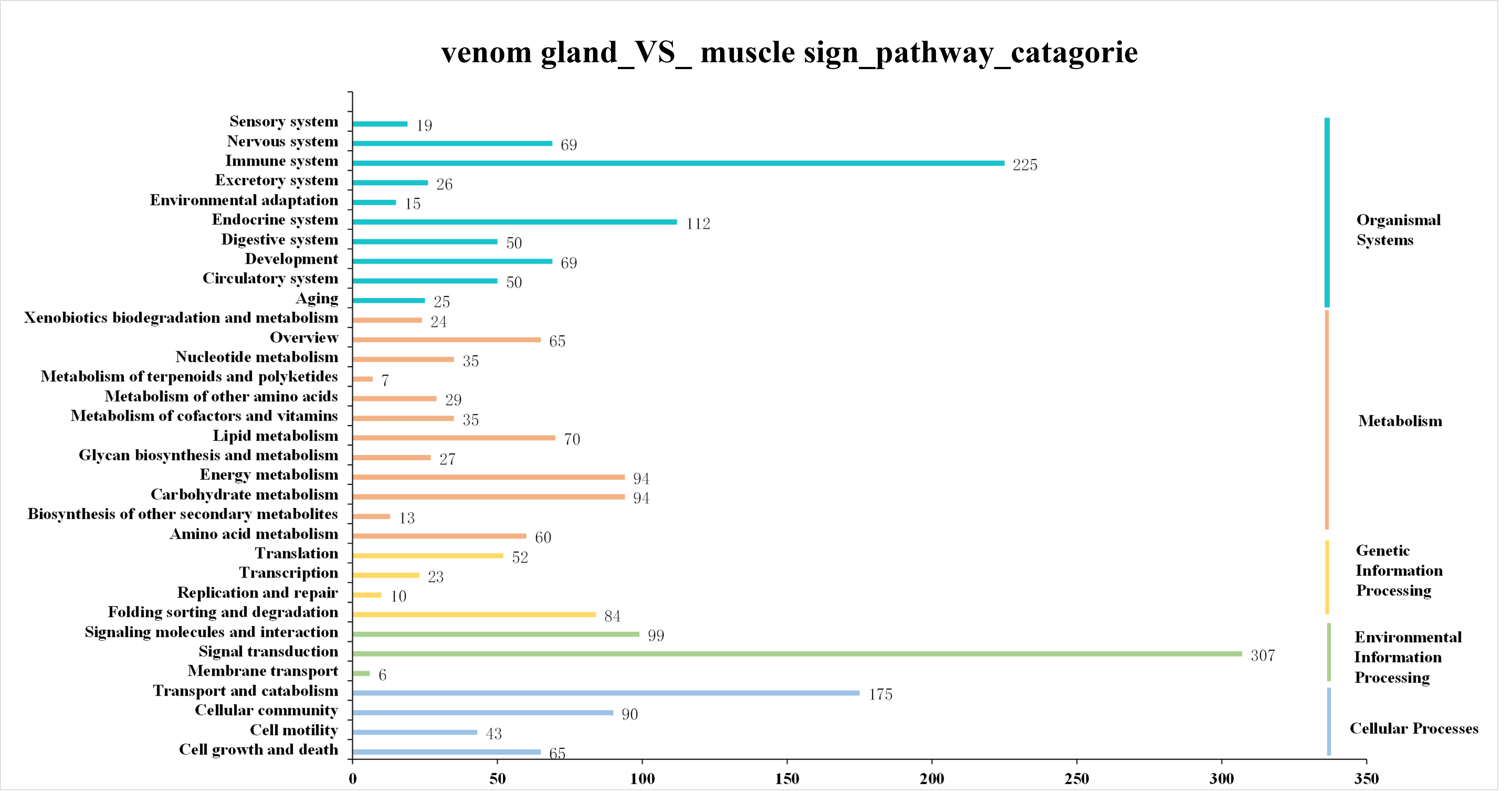
**

**Figure. S3** KEGG annotation of the DEGs in different tissues. Significant enrichment pathways in venom gland vs. muscle comparison.

**
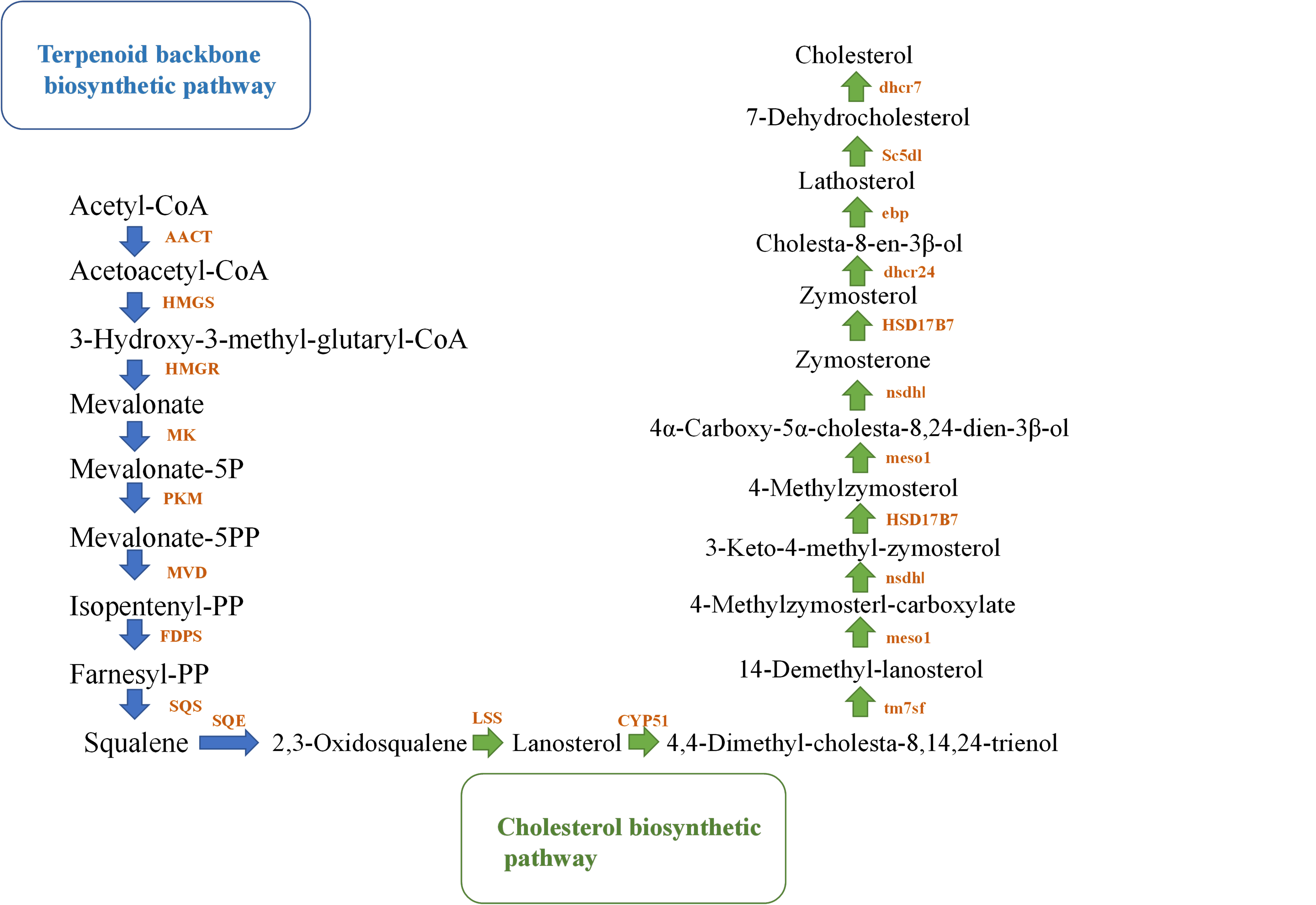
**

**Figure. S4** The enzymes involved in terpenoid backbone biosynthetic pathway and cholesterol biosynthetic pathway.


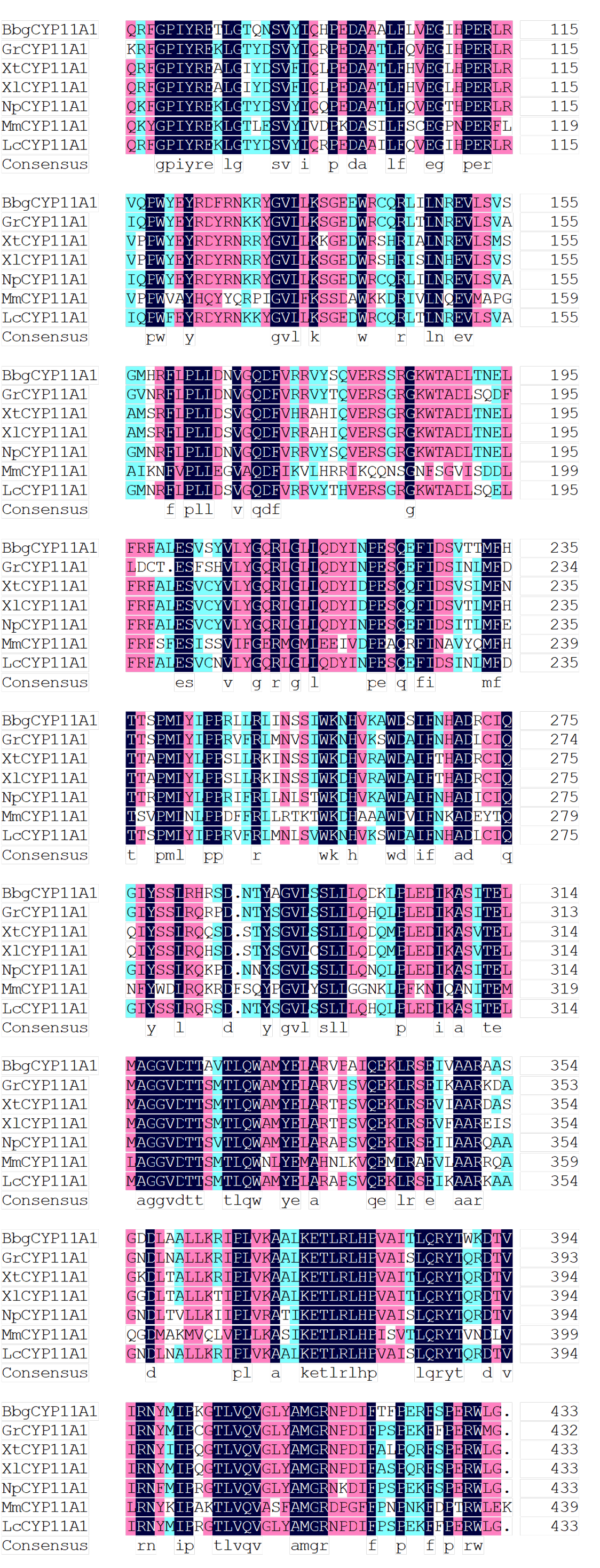


**Figure. S5** Multi-alignment of amino acid sequence of *B. bufo gargarizans* P450scc and its homologs with Glandirana rugose, Xenopus tropicalis,

Xenopus laevis, Nanorana parkeri, Mus musculus, Lithobates catesbeianus.

**Table S1.** Summary of RNA-Seq data for each sample.

| **Iterm** | **Raw reads number** | **Clean reads number** | **Clean bases** | **Average length（bp）** | **Q20（%）** | **Q30（%）** | **GC（%）** |
| --- | --- | --- | --- | --- | --- | --- | --- |
|  |  |  |  |  |  |  |  |
| **A1** | 59,956,616 | 57,304,788 | 8,143,015,956 | 142.10 | 99.12% | 96.95% | 48.36% |
| **A2** | 54,856,540 | 48,627,078 | 6,707,261,479 | 137.93 | 99.08% | 97.12% | 48.94% |
| **A3** | 57,196,624 | 51,063,968 | 6,749,047,541 | 132.17 | 99.14% | 96.96% | 50.49% |
| **B1** | 54,095,132 | 48,045,522 | 6,282,969,490 | 130.77 | 99.16% | 97.21% | 49.93% |
| **B2** | 52,671,266 | 46,756,950 | 6,055,271,245 | 129.51 | 99.16% | 97.23% | 50.14% |
| **B3** | 49,617,698 | 44,062,608 | 5,789,845,969 | 131.40 | 99.17% | 97.22% | 50.11% |
| **C1** | 58,783,898 | 52,243,048 | 7,064,394,255 | 135.22 | 99.15% | 97.15% | 50.03% |
| **C2** | 56,510,558 | 50,731,080 | 6,699,050,954 | 132.05 | 99.17% | 97.21% | 49.80% |
| **C3** | 50,165,774 | 45,161,614 | 6,039,252,199 | 133.73 | 99.15% | 97.16% | 50.03% |

**Table S2.** Annotation summary in different databases.

| **Database** | **Number of genes** | **Percentage (%)** |
| --- | --- | --- |
| Annotated in CDD | 36,891 | 12.22 |
| Annotated in KOG | 30,373 | 10.06 |
| Annotated in NR | 72,301 | 23.95 |
| Annotated in NT | 55,136 | 18.27 |
| Annotated in PFAM | 24,870 | 8.24 |
| Annotated in Swissprot | 57,570 | 19.07 |
| Annotated in TrEMBL | 69,178 | 22.92 |
| Annotated in GO | 62,642 | 20.75 |
| Annotated in KEGG | 8,777 | 2.91 |
| Annotated in at least one database | 86,783 | 28.75 |
| Annotated in all database | 4,141 | 1.37 |
| Total genes | 301,828 | 100 |

**Table S3.** The enzymes may relate to the biosynthesis pathway of bufadienolides.

| **NO.** | **Name of Gene** | **Enzyme** | **EC no.** |
| --- | --- | --- | --- |
| 1  2  3  4 | CYP11A1  3β-HSD  5β-POR  CYP46A1 | cholesterol monooxygenase (side-chain-cleaving)  3 beta-hydroxysteroid dehydrogenase (NHDH)  3-oxo-5-beta-steroid 4-dehydrogenase  cholesterol 24-hydroxylase | EC:1.14.15.6  EC:1.1.1.270  EC:1.3.1.3  EC:1.14.14.25 |
| 5 | CYP39A1 | 24-hydroxycholesterol 7alpha-hydroxylase | EC:1.14.14.26 |
| 6 | HSD3B7 | cholest-5-ene-3beta,7alpha-diol 3beta-dehydrogenase | EC:1.1.1.181 |
| 7 | CH25H | cholesterol 25-hydroxylase | EC:1.14.99.38 |
| 8 | CYP7B | 25/26-hydroxycholesterol 7alpha-hydroxylase | EC:1.14.14.29 |
| 9 | CYP8b1 | sterol 12-alpha-hydroxylase | EC: 14.14.139 |
| 10 | AKR1DI | 3-oxo-5-beta-steroid 4-dehydrogenase | EC:1.3.1.3 |
| 11 | AKR1C4 | 3alpha-hydroxysteroid 3-dehydrogenase / chlordecone reductase | EC:1.1.1.50/1.1.1.357/1.1.1.225 |
| 12 | CYP27A1 | cholestanetriol 26-monooxygenase | EC:1.14.15.15 |
| 13 | SLC27A5 | solute carrier family 27 (fatty acid transporter), member 5 | EC:6.2.1.7 |
| 14 | AMACR | alpha-methylacyl-CoA racemase | EC:5.1.99.4 |
| 15 | ACOX2 | 3alpha,7alpha,12alpha-trihydroxy-5beta-cholestanoyl-CoA 24-hydroxylase | EC:1.17.99.3 |
| 16 | HSD17B4 | (3R)-3-hydroxyacyl-CoA dehydrogenase / 3a,7a,12a-trihydroxy-5b -cholest-24-enoyl-CoA hydratase / enoyl-CoA hydratase 2 | EC: 1.1.1.-4.2.1.107 4.2.1.119 |
| 17 | SCP2 | sterol carrier protein 2 | EC:2.3.1.176 |
| 18 | BAAT | bile acid-CoA:amino acid N-acyltransferase | EC: 2.3.1.65 3.1.2.2 |
| 19 | cbh | choloylglycine hydrolase | EC:3.5.1.24 |
| 20 | BaiaB | bile acid-coenzyme A ligase | EC:6.2.1.7 |
| 21 | BaiA | 3alpha-hydroxy bile acid-CoA-ester 3-dehydrogenase | EC:1.1.1.395 |
| 22 | BaiCD | 3-oxocholoyl-CoA 4-desaturase | EC:1.3.1.115 |
| 23 | BaiE | bile-acid 7alpha-dehydratase | EC:4.2.1.106 |
| 24 | BaiN | 3-dehydro-bile acid Delta4,6-reductase | EC:1.3.1.114 |
| 25 | BaiF | 3-dehydro-bile acid Delta4,6-reductase | EC:1.3.1.114 |

**Table S4.** qRT-PCR Primers used in this study.

| **Primer name** | **Sequence (5ʹ→3ʹ)** |
| --- | --- |
| q-BbgCYP11A1-F | AATCTTCAACCATGCGGATCGT |
| q-BbgCYP11A1-R | GTCCACACCTCCTGCCATCA |
| q-BbgAdx-F | GCCAGTGACCGATGAGGAGAT |
| q-BbgAdx-R | TGCTCATATCCACCGCTTGTCT |
| q-BbgAdR-F | AGCAGGAGCCTCAGCAGTTC |
| q-BbgAdR-R | ACCAACCGCAGCATCTCTTCT |
| q-Bbg-β-actin -F | GAGCTATGAGTTGCCTGATGGACAG |
| q-Bbg-β-actin -R | AATCCTTACGAATATCCACATCACAC |

**Table S5.** Primers used in this study.

| **Primer name** | **Sequence (5ʹ→3ʹ)** |
| --- | --- |
| BbgCYP11A1-3ʹ RACE | CCTTCCTGCCTCTCCTGGACAATGTTGG |
| BbgCYP11A1-F | AAGGAGAAAAAACCCCGGATCCATGATGCTCTCTCGAAGACTGTG |
| BbgCYP11A1-R | AGCTAGCCGCGGTACCAAGCTTTTAGGAGTGTCTTTCTGAACCA |
| BbgAdR-2A-linker-F | GGCGAAGAATTGTTAATTAAGAGCTCTCATCGAGAAACCAACCGCAGCATCTC |
| BbgAdR-2A-linker-R | GCGGGGGACGTTGAGTCCAACCCTGGACCCATGGCTGCTCGGTGGAGCTGGATGTG |
| BbgAdx-2A-linker-F | CAACTTGAGGAGGTCGAAGTTCAGGAGCTGAGAACTCTTGCTCATATCCACCGCTTG |
| BbgAdx-2A-linker-R | AATTCAACCCTCACTAAAGGGCGGCCGCATGGCAGCGGTGACCAGACTCCTCTC |
| Overlap-F | CAGGGTTGGACTCAACGTCCCCCGCCAACTTGAGGAGGTCGAAGTTCAGGAG |
| Overlap-R | CTCCTGAACTTCGACCTCCTCAAGTTGGCGGGGGACGTTGAGTCCAACCCTG |
| MmAdR-F | GCGAAGAATTGTTAATTAAGAGCTCGGAATTCTTGCTCATGTCAACAG |
| MmAdR-R | TTGAGTCCAACCCTGGACCCATGGCTCCTCGCTGCTGGCACTGGT |
| MmAdx-2A-linker-F | GGCGAAGAATTGTTAATTAAGAGCTCTCAGTGGCCCAGCAGCCGCACGAAGAATTGTTAATTAAGAGCTCGGGTCCAGGGTTGGACTCAA |
| MmAdx-2A-linker-R  AaCPR-F  AaCPR-R | ATTCGAATTCATGGCGGCCGCATGGCGGCCGCTCCGGGCGCCCGACTTGACATGAGCAAGAATTCCCAGCTCCTGAACTTCGACCT  aaccctcactaaagggcggccgcATGCAATCGGAGACGATGAAGCTC  gttaattaagagctcagatctTTACCACACATCACGCAGATATCTC |

**Table S6.** The nucleotide sequences involved in this study.

| **Gene** | **Ecoding sequences** |
| --- | --- |
| BbgCYP11A1  (*Bufo bufo gargarizans*)  BbgAdx  (*Bufo bufo gargarizans*)  BbgAdR  (*Bufo bufo gargarizans*)  MmAdx  (Mus musculus)  MmAdR  (Mus musculus)  AaCPR  (*Anemarrhena asphodeloides* Bunge) | ATGATGCTCTCTCGAAGACTGTGTATTCTTCCTTCTTCCACTGGGTTGCTGAACTACCACTCAGTGGTCAGTGAAGGCTCTGCTGTGGCCTCTCACCAAACATCGGCAACTCCTTTGCCTTATGACCAACTGCCTGGTGACTGGAAGAAAGGATGGAGTAGCTTGTACCATTTCTGGAAAAAGGACGGATTTAAGAATATTCATCATCTCATGGTGGAAAATTACCAACGTTTTGGACCTATTTACAGGGAGACTCTGGGAACACAGAACAGTGTCTACATTCAGCATCCAGAGGATGCTGCTGCATTATTCCTTGTGGAAGGGATACATCCTGAACGTCTTCGGGTTCAGCCATGGTATGAGTATCGTGATTTTCGTAATAAAAGATATGGAGTGCTATTAAAGAGTGGAGAAGAATGGCGTTGCCAAAGACTTATACTGAATCGAGA  GGTCCTTTCTGTGTCAGGGATGCATCGCTTCCTGCCTCTCCTGGACAATGTTGGACAGGACTTTGTTCGTAGAGTTTACTCTCAGGTGGAAAGAAGTAGTCGGGGAAAGTGGACAGCTGATCTGACCAATGAGCTCTTTCGATTTGCATTGGAGTCTGTTTCCTATGTGCTTTATGGACAACGTCTTGGCCTGCTGCAAGATTATATAAACCCTGAATCTCAAGAATTCATAGACTCTGTAACTACCATGTTCCACACCACTAGCCCCATGCTTTACATTCCTCCACGGCTTCTGAGGTTAATTAACTCCTCCATTTGGAAAAACCATGTAAAGGCCTGGGATTCAATCTTCAACCATGCGGATCGTTGTATACAGGGTATCTACAGCTCTCTAAGGCATCGCTCTGACAATACATATGCAGGAGTCCTTTCCAGTCTCCTTCTTCAGGATAAGCTTCCACTTGAAGACATTAAAGCTAGTATCACTGAACTGATGGCAGGAGGTGTGGACACGACAGCAGTAACCCTTCAGTGGGCCATGTATGAGCTGGCACGGGTCCCAGCCATCCAGGAAAAGCTTAGGTCTGAAATTGTAGCTGCCAGGGCAGCATCTGGAGATGACTTGGCAGCACTACTGAAGAGAATCCCATTGGTCAAAGCAGCGTTGAAAGAAACTCTGAGACTGCACCCTGTTGCTATCACATTACAGAGATATACCTGGAAGGATACAGTTATCCGCAACTACATGATACCAAAGGGGACTTTGGTGCAAGTTGGACTTTATGCGATGGGACGGAATCCTGATATCTTCACCTTTCCTGAAAGGTTCTCTCCCGAGCGCTGGCTAGGGCAAGAATCCACTCATTTTAGAGGCTTAAGCTTTGGTTTTGGTCCTCGACAGTGTCTTGGCAGAAGAATAGCAGAAATGGAGATGCAGCTTTTCCTCGTCCATGTTCTGGAGAATTTCAAGATAGAGACAAACCGAATGGTTGAAGTTGAATCAACTTTTAATCTCATTCTCTTCCCTACCAGACCCATTCAGTTAACCTTGTCCCCGTTATCACTGAATGGTTCAGAAAGACACTCCTAA  ATGGCAGCGGTGACCAGACTCCTCTCCGGCTCGCGCTGCCTTCTGGCCAGGTCCAGGAGCGGCATGTCCGCGGTGCGGCGGCCGCTGAGGGCGGCTGGAGGCTGCCTGTGGACCGGGGCGGCGCGGCTCTTCAGCTCAGAAGACAAAGTTACAGTGAAATTTATAAATCGTGATGGAGAAACCATTGTAGCAGAGGGAAAGGTTGGAGAATCCCTTCTGGACCTTGTTGTAGCCAAAAACTTAGACATAGATGGTTTTGGTGCTTGTGAGGGGACGCTGGCCTGTTCTACTTGTCATCTCATCTTCGAGGATCATATATTCCGACAGCTGGAGCCAGTGACCGATGAGGAGATGGATATGCTGGATCTCGCCTATGGACTCACTGAAATGTCAAGACTGGGCTGCCAAATCTGCCTCCAGCCGTTCATGAATGGCATGACTGTGAAAGTTCCCGAGGCGGTGGCAGACGTCAGACAAGCGGTGGATATGAGCAAGAGTTC  TTGA  ATGGCTGCTCGGTGGAGCTGGATGTGGCGCCTTCCTCGCTGCCCTCTCCTCGGCAGGAATCTTCCCGGGACTGGAAGACTGGGTGTAGAACGAAGACTTTCTTCCAAGTCGCAGACACCCCAGATCTGCATAGTTGGCAGCGGCCCAGCCGGATTTTACACCGCGCAGCACCTGTTGAAGCACCACACACAAGCCGTAGTTGATATTTATGAGAAGCTGCCTGTACCATTTGGCCTGGTCCGATTTGGCGTGGCACCAGATCATCCTGAAGTCAAAAATGTTATTAACACTTTTACACAAACTGCCAAATCTGAGCGCTGCAACTTCTTAGGAAATGTGACAGTCGGAAGAGATGTAACCGTCGAGGAGCTGCAGGAAGCCTATCATGCTGTAGTGCTGAGCTATGGAGCAGAAGATAAGAGGGAGCTGGAGATCCCCGGAGAGCAGCTTCAAGGGGTATATTCAGCCAGAGACTTTGTCGGCTGGTACAATGGACTTCCAGACAATAGACATTTGTCCCCAGATCTGAGCAGCGAGACGGCTGTCATCTTAGGACAAGGAAATGTTGCACTGGACATTGCAAGAATGCTGCTTTCCCCTCTTGAACTGTTAAAGAAAACCGACATCGCACAACCTGCTCTTGAAGCCTTATCCCAAAGTCGCGTCAAGAAAGTCTGGCTGATTGGACGTCGTGGACCTCTGCAGGTGGCATTCACCATAAAGGAATTACGAGAGATGATTAACCTGCCAGGAACTCGTACGATCAATGACCCTTCTGATTTCCAAGGCCTTGGGGACATTTTAAAAGACCTCCCTAGGCCAAGGAAACGACTTACAGAACTGCTGGTAAAGTCAGCACTGGAGACGCCTGGAGATAAAGAGGCCGCTCGTAGGGCTCAGTGTAGCAGAGAATGGGGTCTGCGTTTCTTCCGCAGCCCGGTCGCCGTGCTTCCCAGTGAAGATGGAAAAAGAGCAGCTGGGATCCGGCTATCGGTCACTCGACTTGAGGGATCTGGGGAGAGTACAGTGGCTGTTCCTACAGGAGAAACAGAAGATGTATCTTGTGGCCTTATTTTCAGCAGCATTGGGTATAGAAGTGTCCCTATCTCTCCAGGTGTGCCTTTCCTTCCCAAGCAGGGCATCATTCCAAATAATTTGGGAAGGGTGCACGGGGAGCCAGGACTGTACTGCAGCGGCTGGGTGAAAAGGGGTCCCACAGGTGTCATCACAACTACAATGACAGACAGTTTTGACACAGCACAGGCTTTGATGGAGGACATGAAGTCGGGGGCATTGAAGTTGTCAGATTCTCGAGCAGGAGCCTCAGCAGTTCGAGAGCTTCTTCTTCTCAGAGGAGTTCAAACTGTCTCCTTCTCAGACTGGGAAAAGATAGATGCGATGGAGACAGATCAAGGTGAGAAGGTGGGAAAACCTCGAGAAAAGATTTTAGACACAGAAGAGATGCTGCGGTTGGTTTCTCGATGA  ATGGCGGCCGCTCCGGGCGCCCGACTCCTGCGCGCGGCCTGCGCCTCCGTCCCTTTCCGCGGCCTTGACCGCTGTCGGCTGCTGGTCTGCGGGACCGGAGCGGGAACTGCCATCTCTCCGTGGACCCCGAGTCCCCGCCTGCATGCAGAGGCCGGGCCCGGCCGGCCGCTGAGCGTGTCTGCGCGCGCGCGGAGCAGCTCAGAAGATAAGATAACAGTCCACTTCAAGAACCGAGATGGCGAGACGCTAACGACCAAGGGGAAAATTGGCGACTCTCTGCTAGATGTTGTGATTGAGAACAACTTAGATATCGATGGGTTTGGTGCGTGTGAGGGAACGTTGGCTTGCTCTACTTGTCATCTTATCTTTGAGGATCACATCTATGAGAAGTTAGATGCCATTACTGATGAAGAGAATGACATGCTTGACCTGGCTTTTGGACTAACAGACAGGTCAAGGTTGGGCTGCCAAGTTTGTCTGACCAAGGCTATGGACAATATGACTGTGCGTGTGCCTGAAGCAGTGGCGGATGTCCGACAGTCTGTTGACATGAGCAAGAATTCCTAA  ATGGCTCCTCGCTGCTGGCACTGGTGGCGCTGGTCCGCGTGGTCTGGGCTTCGGCCGTCTCCCTCCAGGAGCACTCCGACCCCAGGCTTCTGCCAGAAGTTCTCCACACAGGAGAAGACCCCTCAGATCTGTGTGGTCGGCAGTGGCCCAGCTGGCTTCTACACAGCCCAACACTTGTTAAAGCACCACACCCATGCCCACGTAGACATCTACGAGAAGCAGCTCGTGCCATTCGGCCTGGTGCGCTTTGGTGTGGCACCTGACCATCCTGAAGTAAAGAATGTTATCAACACATTTACACAGACAGCCCGCTCAGACCGCTGTGCCTTCCAGGGCAATGTGGTGGTGGGCAGGGACGTGTCGGTTCCAGAGCTTCGGGAAGCCTACCATGCTGTGGTGCTGAGTTATGGAGCAGAGGACCACCAACCCCTGGGAATTCCTGGCGAGGAGCTGCCTGGAGTGGTCTCAGCCCGGGCCTTTGTGGGCTGGTACAATGGACTTCCCGAGAACCAGGAGCTGGCGCCAGATCTGAGCTGTGACACGGCTGTAATTCTGGGACAGGGGAATGTGGCTCTGGATGTGGCCCGGATCCTGCTGACCCCACCTGAGCACCTGGAGAAAACAGACATCACAGAGGCTGCATTGG  GGGCCCTGAGGCAGAGTCGGGTGAAGACTGTGTGGATAGTGGGCCGGCGTGGGCCCTTGCAAGTAGCGTTCACCATTAAGGAGCTTCGGGAGATGATTCAGTTGCCAGGAACCCGGCCCATTTTGGATCCTTCGGATTTCTTGGGCCTCCAGGACAGAATTAAGGATGTCCCCCGTCCAAGGAGGCGGCTAACAGAACTGCTGCTTCGGACAGCCACGGAGAAGCCAGGAGTGGAAGAGGCTGCCCGCCAGGCACTGGCCTCCCGGGCCTGGGGTCTCCGCTTTTTCCGAAGCCCCCAGCAGGTGCTCCCTACCCCAGATGGCCAACGGGTAGCAGGCATCCGCCTGGCAGTTACTAGTCTAGAGGGTGTTGGGGAGTCCACTCGGGCAGTGCCCACAGGAGACGTGGAGGACCTCCCTTGTGGACTGCTGCTGAGCAGCGTTGGGTATAAGAGCCGCCCCATCGACCCCAGCGTGCCCTTTGACCCCAAGCTTGGAGTCATCCCCAACACAGAGGGCCGGGTTGTGAATGTCCCAGGCCTCTACTGCAGTGGCTGGGTGAAGAGGGGACCCACAGGTGTCATCACCACAACCATGACAGACAGCTTCCTCACCAGCCAGGCGCTGCTGGAGGACCTGAAGGCGGGGCTGCTGCCCTCCGGCCCCAGACCCGGCTATGTGGCCATTCAAGCCCTGCTCAGCAATCGAGGAGTCCGGCCAGTGTCTTTCTCAGACTGGGAGAAGCTGGATGCTGAGGAAGTCTCTCGAGGCCAAGGTACTGGGAAACCAAGGGAGAAGCTGGTGGATCGAAGAGAGATGCTGCGGCTGCTGGGCCACTGA  ATGCAATCGGAGACGATGAAGCTCTCACCTTTGGATCTGATGATGGCGATCTTGACCGGGAAGCTCGGGGACAGCGGCCTACCCCCAGAGGTGGCCTCGATCACCGAGAACCGCGAGCTCCTTATGATTCTGACGACGTCGATCGCCGTCCTGATTGGATGCGCTGTTGTTTTCTTGTGGCGGCGGTCGAGCGGGAAGTCCAGCAAGTCTGTGGAACCTCCGAGACCTCTCGTGATTCCCAAGGAGCCGGAGCCTGAGGTCGACGATGGGAAGAAGAAAGTCACCATCTTTTTTGGTACCCAGACCGGGACGGCTGAAGGTTTTGCTAAGTCGCTGGCGGAAGAGGCCAAGGCTAGATATGACAAGGCGACCTTCAAAGTCGTCGATCTGGATGATTACGCGGCGGACGATGATGAGTACGAGGAGAAGATGAAGAAAGAAACCCTAGCCCTGTTCTTCTTGGCAACATATGGAGACGGGGAACCAACTGATAACGCTGCCAGATTCTATAAATGGTTTACTGAGGGGAAAGAGAGGGAACAATGGCTGGAGAATCTTCAATATGCTGTGTTCGGTTTGGGCAATAGGCAGTACGAGCATTTCAATAAGGTGGCAAAGGTGGTGGATGAGGTCCTTGCTGAACAGGGAGCAAAGCGCCTTGTCCCAGTGGGTCTTGGGGATGATGATCAGTGCATTGAGGATGATTTCGCCGCATGGAGAGAGCTATTATGGCCAGAATTAGATCAATTGCTACGAGATGAAGATGATGCATCGGGTGCATCCACCACATATACAGCTGCTGTTCCTGAATACCGAGTTGTATTGATTGACTCTGCAGGCGCATCTCATTTGGAGAAGAATTGGAGTCTTGCAAATGGTCATGCTGTTCATGATATTCACCATCCATGCAGAGCTAATGTGGCTGTGCGGAGGGAGCTTCATACTCCAGCTTCTGACCGTTCCTGCATTCATCTGGAGTTTGATATTTCGGGGACAGGTCTTGCATATGAAACAGGGGATCATGTTGGTGTATATTCAGAGAATTGTCTTGAGACTGTAGAGGAGGCAGAGAAGTTATTAGGTCTTCCATCAGACACATTTTTCTCCATTCATGCTGACAATGAAGATGGAACTCCACTTGGCAGCTCTTTGCCACCTCCATTCCCATCTCCATGCACTTTAAGAACAGCACTCACACGCTATGCTGATCTTCTGAATTCTCCTAAAAAGGCTGCTTTGGTTGCTTTAGCTACTCATGCTTCTGATCCCAATGAAGCAGAAAGATTGAGATTTTTGGCTTCTCCTGCAGGAAAGGATGAGTACTCTCAATGGGTAGTTGCTAGTCAGAGGAGCCTTCTGGAGGTTATGGCTGAGTTTCCTTCAGCCAAGCCTCCACTAGGAGTTTTCTTTGCAGCAATAGCCCCCCGCTTGCAGCCTAGATATTATTCAATATCATCTTCACCAAGGATGGCACCAACTAGAATTCATGTCACATGCGCTCTAGTTTATGGACCAACACCTACAGGAAGGATTCACAAAGGAGTCTGCTCAACCTGGATGAAGCATGCAGTTCCTTCAGAGGAGAGCAAAGAGTGCAGCTGGGCCCCTATATTTGTGAGACAGTCAAACTTCAAACTCCCTTCACATCCCTCCACACCAATTATTATGATTGGCCCAGGCACAGGGTTGGCACCCTTCAGGGGCTTCCTGCAGGAAAGATTGGCACTAAAGCAAGCTGGCACAGAACTTGGCCCTGCCATTCTCTTCTTTGGTTGCCGGAACCGAAGAATGGATTTCATATACGAGGATGAGCTGAAAAATTTTGTTGAGGAAGGTGCACTTTCTGAGCTGATTGTAGCCTTTTCTCGTGAGGGTCAGACCAAGGAATATGTGCAGCATAAGATGGCTGAGAAGGCTGTTGAGCTCTGGAATATCATCTCTAAGGGTGGATATCTTTATGTATGTGGTGACGCGAAAGGTATGGCTAGAGATGTCCATAGAGTGCTCCACACTATAGTTCAAGAGCAGGGATCATTGGATAGCTCAAAGGCTGAGAGCATGGTAAAGAGTCTACAGATGGAAGGGAGATATCTGCGTGATGTGTGGTAA |

**Table S7.** Plasmids used in this study.

| **Plasmids** | **Description** |
| --- | --- |
| pESC-Leu | Yeast expression vector |
| pESC-Leu-AaCPR | Gene CPR from *A. asphodeloides* Bunge was synthesized and cloned into pESC-Leu |
| pGL01 | CTL1 was cloned into pESC-Leu |
| pGL02 | CTL2 was cloned into pESC-Leu |
| pGL03 | Gene BbgCYP11A1 from *B. bufo gargarizans* was synthesized and cloned into pESC-Leu |
| pGL04 | Gene CYP11A1 from *B. bufo gargarizans* was synthesized and cloned into pESC-Leu-AaCPR |
| pGL05 | Gene BbgCYP11A1 from *B. bufo gargarizans* was synthesized and cloned into pGL01 |
| pGL06 | Gene BbgCYP11A1 from *B. bufo gargarizans* was synthesized and cloned into pGL02 |
